# Selection of Microsatellite Markers for Bladder Cancer Diagnosis without the Need for Corresponding Blood

**DOI:** 10.1371/journal.pone.0043345

**Published:** 2012-08-22

**Authors:** Angela A. G. van Tilborg, Lucie C. Kompier, Irene Lurkin, Ricardo Poort, Samira El Bouazzaoui, Kirstin van der Keur, Tahlita Zuiverloon, Lars Dyrskjot, Torben F. Orntoft, Monique J. Roobol, Ellen C. Zwarthoff

**Affiliations:** 1 Department of Pathology, Erasmus MC, Rotterdam, The Netherlands; 2 Aarhus University Hospital, Department of Clinical Biochemistry, Skejby, Denmark; 3 Department of Urology, Erasmus MC, Rotterdam, The Netherlands; National Cancer Center, Japan

## Abstract

Microsatellite markers are used for loss-of-heterozygosity, allelic imbalance and clonality analyses in cancers. Usually, tumor DNA is compared to corresponding normal DNA. However, normal DNA is not always available and can display aberrant allele ratios due to copy number variations in the genome. Moreover, stutter peaks may complicate the analysis. To use microsatellite markers for diagnosis of recurrent bladder cancer, we aimed to select markers without stutter peaks and a constant ratio between alleles, thereby avoiding the need for a control DNA sample. We investigated 49 microsatellite markers with tri- and tetranucleotide repeats in regions commonly lost in bladder cancer. Based on analysis of 50 blood DNAs the 12 best performing markers were selected with few stutter peaks and a constant ratio between peaks heights. Per marker upper and lower cut off values for allele ratios were determined. LOH of the markers was observed in 59/104 tumor DNAs. We then determined the sensitivity of the marker panel for detection of recurrent bladder cancer by assaying 102 urine samples of these patients. Sensitivity was 63% when patients were stratified for LOH in their primary tumors. We demonstrate that up-front selection of microsatellite markers obliterates the need for a corresponding blood sample. For diagnosis of bladder cancer recurrences in urine this significantly reduces costs. Moreover, this approach facilitates retrospective analysis of archival tumor samples for allelic imbalance.

## Introduction

Microsatellite analysis utilizes short, highly polymorphic repeated sequences within the genome. The number of repeats forming a specific microsatellite often varies between the maternal and paternal allele. Microsatellites are used to determine allelic imbalance (AI) or loss-of-heterozygosity (LOH) at particular loci in tumor genomes and can be used as a marker for the presence of tumor cells. To this purpose microsatellite analyses compare the intensity of amplification products of the paternal and the maternal allele from a tumor sample against the ratios from a corresponding normal control (for example leukocytes). When the microsatellite marker is informative (the length of the two alleles differs) the amount of product from both alleles will be the same. On a capillary sequencer this is displayed as two peaks of about equal height. LOH or AI of a chromosomal region in cancer is usually concluded when the ratio between the allele peaks in tumor DNA is smaller than 0.5–0.7 or over 1.5–2 when compared to the control [1], [2], [3], [4].

Bladder cancer (BC) is the fifth most common malignancy in the Western world after breast, prostate, colorectal and lung cancer [5]. More than 70% of primary BC manifest as low grade, non-muscle invasive (pTa, pT1) tumors. After removing these tumors by transurethral resection (TUR), the recurrence rate is high (70%) and many patients will develop multiple metachronous recurrences [6]. Progression from a non-muscle invasive (NMIBC) to a muscle invasive cancer (MIBC) occurs in 10–20% of cases, especially if the original tumor was of high grade [7], [8].

At present, the standard procedure for diagnosing BC is cystoscopy. Cystoscopy is an invasive diagnostic approach that is unpleasant for the patient, who has to undergo such controls every 3–12 months for many years after resection of the primary tumor. We estimate that between 1–2 million cystoscopies are being carried out per year in EU and USA for follow up of these patients. Unfortunately, the cytological examination of cells present in voided urine alone does not provide a safe screening alternative for cystoscopy because of its low sensitivity, especially for the detection of low-grade tumors [9]. Similarly, current molecular urine based assays such as fluorescent in situ hybridization (FISH), NMP22 and BTA have sensitivities that are low for the detection of mostly low grade and stage recurrent BC [10], [11]. As a result, a number of different molecular analyses on DNA isolated from cells present in voided urine samples have been developed with the goal to improve the sensitivity of detection and to reduce the frequency of invasive cystoscopy examinations. One of those assays involves the detection of mutations in the *FGFR3* gene [12]. Mutations in this gene are very frequent in pTa bladder tumors (up to 75% [13], [14]). However, this assay is not an option for detecting tumors without a mutation in this gene, especially since for patients with an *FGFR3* wild type primary tumor, the frequency of *FGFR3* mutations in recurrences is much lower than for patients with an *FGFR3* mutant primary tumor (19% and 81%, respectively) [15].

Many tumors display genomic alterations like gene mutations and numerical aberrations affecting short genomic regions to entire chromosomes. These tumor-specific genomic alterations can be detected by molecular techniques such as FISH [16], [17] or by microsatellite analysis (MA). Assays detecting these alterations are proving to be particularly helpful in the identification of cancer patients via noninvasive or little-invasive methods [18], [19], [20], [21]. In bladder cancer for instance, losses of parts of or total chromosome 9 are commonly observed as they arise early in the development of the tumor [22]. Progression of the disease is usually accompanied by additional numerical alterations involving chromosome 8p, 10, and 17p [23], [24], [25]. We and others have previously shown that the detection of recurrent bladder cancer can be improved by microsatellite analysis of in cells obtained from voided urine samples [4], [26].

During this work we observed that the PCR products from microsatellites with dinucleotide repeats often had multiple (stutter) peaks due to dissociation of the DNA strands and aberrant reannealing. In addition, many microsatellite markers had aberrant ratios between peak heights in control DNA, possibly due to copy number variations in the genome. Moreover, the need to analyze control blood DNA made the microsatellite assay (MA) expensive [27]. To address these problems more systematically, we have selected tri- and tetranucleotide repeats in genomic regions commonly affected in bladder cancer and designed primers around these repeats for amplification [28]. These new microsatellite markers were then tested for constant peak height ratios and technical performance (i.e. no stutter peaks, fair amplification) in a series of blood DNA samples. The 12 best performing markers were subsequently analyzed in urine DNAs from healthy individuals to determine the cut off values of the ratio of the peak heights in order to obtain a specificity of 95%. Finally, we validated the markers in urine DNAs from patients diagnosed with a recurrent bladder tumor.

## Materials and Methods

### Samples

Blood samples were collected from 50 patients with bladder cancer. Urine samples were collected from 106 individuals without history of neoplastic disease. These tumor-negative samples were derived during a screening study in elderly males (over 50 years of age) without previous signs of bladder cancer [29]. DNA from 104 primary tumors was available for LOH analysis. All tumors were non-muscle-invasive (89% Ta, 11% T1). All tumors were grade 1 or 2. Urine samples from patients scheduled for TUR were collected before resection of a histologically proven recurrent tumor from 102 patients. Informed written consent was obtained from all patients, and research protocols were approved by institutional review boards or ethical committees in the two involved countries (the Central Denmark Region Committees on Biomedical Research Ethics Denmark and the The Medical Ethical Committee of the Erasmus MC (METC), the Netherlands). Clinicopathological data for these samples is given in Table S1.

### DNA extraction and LOH analysis

After collection, the urine was checked for the amount of leukocytes, erythrocytes and nitrite with a dipstick (Siemens Multistix® 10 SG). Cells were pelleted by centrifugation at 3,000 rpm for 10 minutes at 4°C. Cell pellets were washed twice with 10 ml PBS, resuspended in 1 ml PBS, transferred to an Eppendorf vial, and collected by centrifugation for 5 minutes at 6,000 rpm. Supernatant was discarded and the cell pellet was stored at −20°C until DNA isolation. DNA was extracted from blood, tumor tissue (formalin fixed paraffin embedded (FFPE)) and urine and purified with appropriate kits (Qiagen, Hilden, Germany) following instructions of the manufacturer. Concentrations of DNA were measured with a Quant-iT PicoGreen® dsDNA Assay Kit (Molecular Probes, Leiden, The Netherlands). PCR of different microsatellite markers was performed with separate primer pairs of which 1 oligonucleotide was labeled at the 5-end with the fluorescent dyes 6-FAM (Invitrogen). Amplification of specific DNA was done in a reaction volume of 15 µl including 0.2 mM dNTPs, 2.5 mM MgCl2, and 0.5 U AmpliTaq. Cycling was performed with a Biometra thermocycler using the following temperature conditions: 95°C for 5 min, 28 cycles at 95°C for 45 s, 55°C for 45 s, and 72°C for 45 s, followed by a final extension step of 10 min at 72°C. The PCR products were subsequently denatured for 1 min at 95°C in HiDi formamide (Applied Biosystems) and separated on an ABI PRISM 3100 Genetic Analyzer equipped with a 36 cm capillary array loaded with POP-7 polymer. 500-Liz was used as internal size standard (Applied Biosystems). Analysis of the samples was carried out with the GeneMarker software version 1.7 from SoftGenetics (State College, PA).

### Statistical analysis

The Statistical Package for the Social Sciences 18 (SPSS, Inc.) was used for data analysis. Sensitivity, specificity, and predictive values were determined for every marker and for all markers. Diagnostic accuracy for the model with methylation markers as determined by AUC (Area under the curve). Results were considered statistically significant at p<0.05.

## Results

### Selection of microsatellites

**Figure 1:**
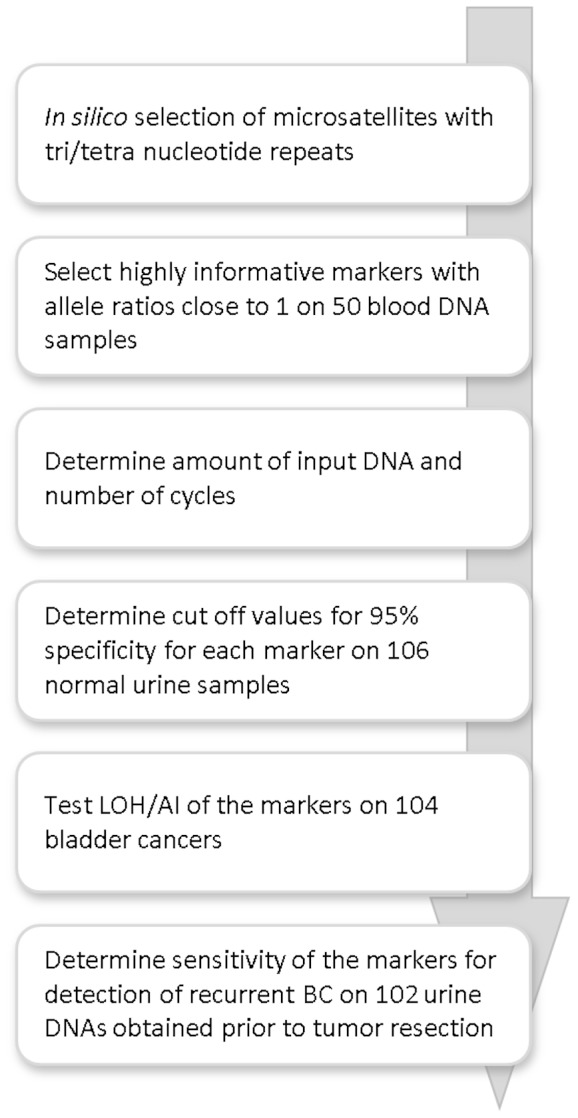
Flow chart of the study.

**Figure 2:**
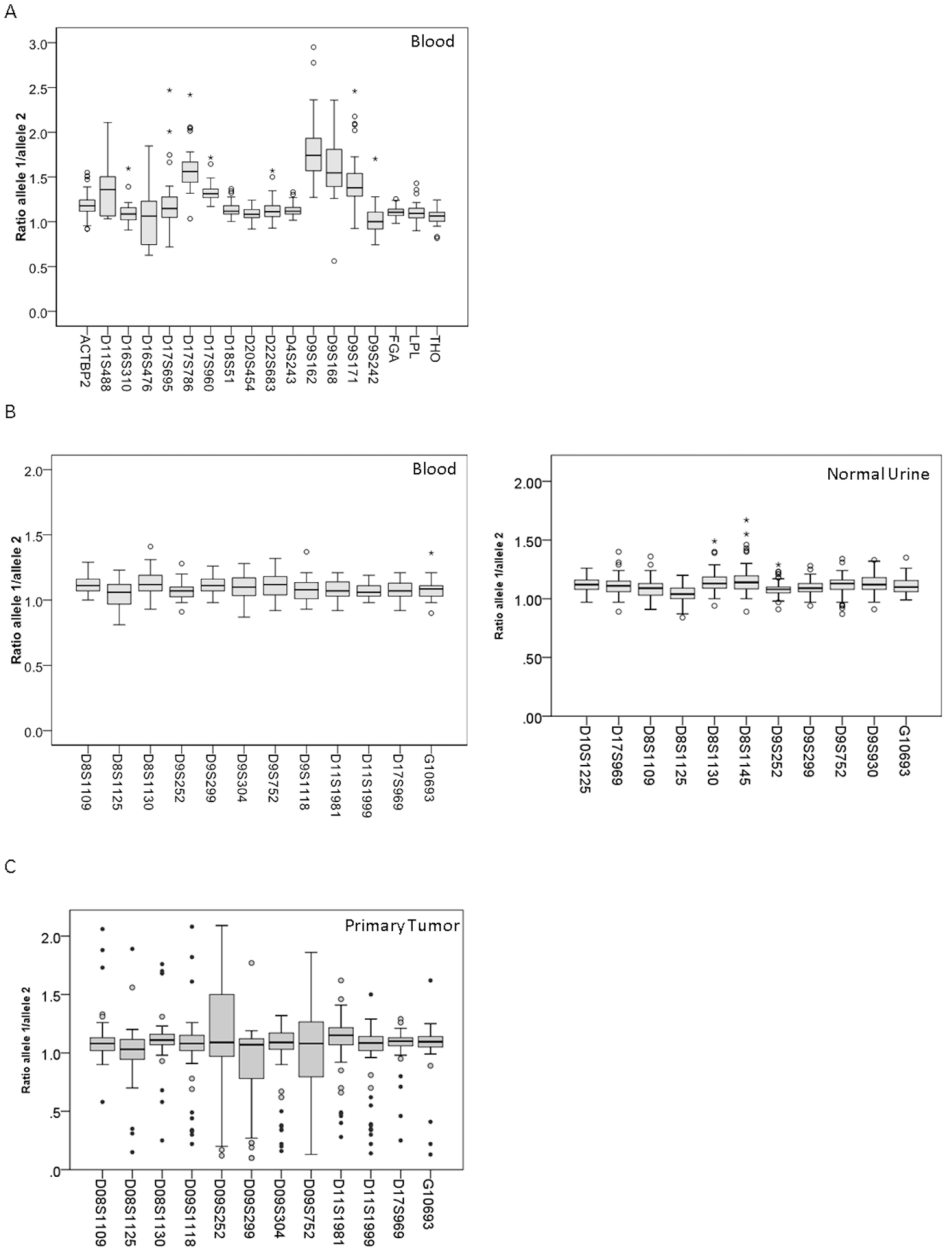
Overview of the variation between allele ratios for different markers. On the Y-axis, the ratio between the two alleles is given. On the X-axis, the different microsatellite markers are listed. A. The boxplots show that some previously used markers have a large variation in their allele ratio based on an analysis of blood DNA samples from 50 individuals. B. Behavior of the 12 selected markers, indicating they have very little variation in their allele ratio when tested on normal blood and urine from healthy individuals. C. In primary tumor DNA the allele ratio is much more variable due to LOH/AI.

Our project consisted of different phases (Figure 1). We previously used a group of 20 microsatellite markers for detection of recurrent bladder cancer in voided urine of patients under surveillance for possible recurrent tumors [4], [18], [30], [31], [32]. However, with several of these markers assessing allele ratio was difficult due to stutter peaks. Also, these markers did not accurately cover the most interesting areas of LOH in bladder tumors. In addition, in many patients the allele ratio in control DNA varied possibly due to genomic copy number variations as displayed in Figure 2A. Based on this experience we decided to select a new panel of microsatellite markers with a relatively constant ratio between alleles thus obliterating the need for a control blood DNA sample. To reduce the chance of stutter peak formation, we selected 49 tri- and tetranucleotide repeat-containing microsatellites from the UCSC genome browser (http://genome.ucsc.edu), and the Généthon panel (http://cedar.genetics.soton.ac.uk/pub) in regions of chromosomes 8, 9, 10, 11 and 17 that display LOH in bladder cancers [28]. The microsatellite markers had to have a minimum level of heterozygosity of 65% and a fragment length between 100–300 bp to reduce amplification difficulties of sheared DNA.

### Technical reproducibility and cut off values for each marker

**Figure 3:**
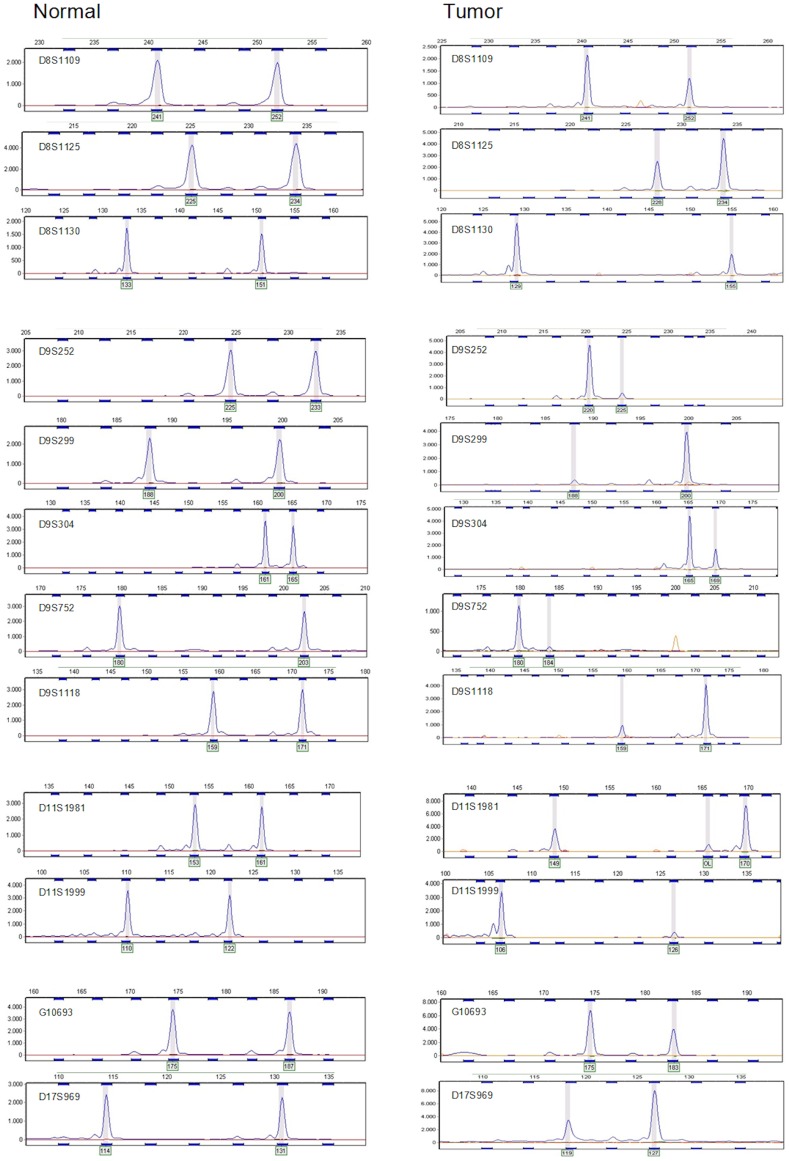
Examples of the electropherograms for the selected markers, ordered to their chromosomal position. On the Y-axis, the peak intensity is given. On the X-axis, the fragment size is given in basepairs. On the left side, results from normal tissue are shown. Note that these markers have few or no stutter peaks and a fairly constant ratio (close to 1) between the heights of the two alleles. On the right side, results from representative tumor samples with LOH are shown.

The markers were then tested on 50 blood samples and 12 microsatellites markers with the highest percentage of heterozygosity and a peak ratio between alleles that was close to 1 were selected for further study (Table 1, Figure 2B). Information about the other 37 markers is given in Table S2. Figure 3 shows electropherograms of the markers. We determined the best settings for reproducibility by varying input DNA concentration and number of cycles. Based on this we chose to use 5–10 ng of input DNA in 28 PCR cycles for subsequent experiments. To determine the cut off values such that each marker would be 95% specific in a diagnostic urine test, we assayed them in duplicate on urine DNAs from 106 non-cancer controls of 50 years and older. The 12 markers with their upper and lower cut off values are listed in Table 2. Table 2 also shows that the standard deviations are below 10%.

**Table 1:** Microsatellite markers selected for this study.

Marker	Size	Locus	Het	F/R	Sequence
D8S1109	143–167	8p	0.88	F	TCAGAATTGCTCATAGTGCAAGA
				R	ACTGTCTTGGTACATTTGTTTACCC
D8S1125	221–233	8p	0.69	F	CCCCCTAAAATTTAGCTCCA
				R	TATGCCTAGCCCTCCTTTCT
D8S1130	128–148	8p	0.94	F	GAAGATTTGGCTCTGTTGGA
				R	TGTCTTACTGCTATAGCTTTCATAA
D9S252	152–176	9q	0.75	F	CAAATTTGGCCTTGAACCAT
				R	AGCCCAGATATCCCCAAGTT
D9S299	178–198	9q	0.70	F	AAGTGTTGCATCAGAGCCTC
				R	AGTGTGAACATTATTTCATTCTGG
D9S304	135–175	9q	0.86	F	GTGCACCTCTACACCCAGAC
				R	TGTGCCCACACACATCTATC
D9S752	178–201	9q	0.73	F	CAGAGGTTGCAGTGAGCTA
				R	GCAAAGTCAGGCCATTATAC
D9S1118	141–177	9q	0.79	F	CAGGATATTATGTGATGGAATCC
				R	CTGCTGACTCCAAAAATATGC
D11S1981	134–178	11p	0.85	F	AATTCCTTTACTCCAGAAAGG
				R	CAGATTTCTGCTTTCCCAGA
D11S1999	109–137	11p	0.78	F	TACATGGCAGCAGGCATATA
				R	GAGTAAACAAGATTGCTAGATAGGC
D17S969	111–132	17q	0.73	F	ATCTAATCTGTCATTCATCTATCCA
				R	AACTGCAGTGCTGCATCATA
G10693	174–194	17p	0.94	F	ACATACAGCACAGGCCAAAT
				R	CCAGTCTTCCGTCACTATGC

Het: the expected heterozygosity (%) according to the CEPH database (http://www.cephb.fr/en/cephdb/browser.php). F/R: forward or reverse primer.

**Table 2:** Upper and lower borders set at 95% specificity based on urine samples from healthy individuals.

Marker	Av Ratio	Stdev	95% interval
D8S1109	1.09	0.08	0.93–1.29
D8S1125	1.04	0.08	0.85–1.19
D8S1130	1.15	0.09	1.00–1.40
D9S252	1.08	0.06	0.97–1.23
D9S299	1.10	0.07	0.95–1.27
D9S304	1.10	0.11	0.86–1.33
D9S752	1.12	0.08	0.92–1.31
D9S1118	1.10	0.10	0.97–1.42
D11S1981	1.14	0.09	0.93–1.39
D11S1999	1.12	0.09	0.93–1.45
D17S969	1.11	0.08	0.95–1.33
G10693	1.11	0.07	1.00–1.29

### Performance of the microsatellite assay in tumors

Subsequently the markers were tested on DNA from the primary bladder tumors of 104 patients. Ninety-two tumors were pTa, 11 were pT1, and of 1 tumor no stage information was available. Twenty-four tumors were grade 1, 79 grade 2, while grade information was missing in 1 case. LOH or allelic imbalance was defined when the ratio of the peaks was higher than the upper border or lower than the lower border as indicated for each marker in Table 2. LOH for one or more markers was found in 59 tumors (57%), while 45 tumors did not have LOH for any of the markers tested. The most frequently lost markers were all on chromosome 9, D9S299, D9S252, and D9S752 (LOH in 29–37% of all samples) (Table 3). Any LOH was found in 53/92 (58%) pTa, 6/11 (55%) pT1 and in 12/24 (50%) G1 and 47/79 (60%) G2 tumors. The allele ratios in tumor DNA were much more variable than in normal blood or urine DNA, due to LOH/AI (Figure 2C).

**Table 3:** Observed heterozygosity and percentage LOH/AI in tumor samples for each marker.

	Total	Het%	LOH/AI%
D8S1109	103	66	9
D8S1125	104	70	11
D8S1130	104	86	13
D9S252	103	77	30
D9S299	104	74	29
D9S304	103	82	18
D9S752	103	80	37
D9S1118	103	86	25
D11S1981	103	88	17
D11S1999	102	87	15
D17S969	104	71	9
G10693	104	72	9

### Determination of the sensitivity of the markers to detect recurrences in urine-derived DNA

Subsequently, we determined the sensitivity of the markers for the detection of recurrent tumors in the same patients of whom we analyzed the primary tumor. A total of 102 urine samples were available, obtained before resection of a recurrent tumor in these patients. Microsatellite assays were performed in duplicate. LOH was assumed when the allele peak ratio of both tests was outside the cut off values shown in Table 2. Markers D9S752, D9S252, D9S304, D9S299 and G10693 displayed LOH in approximately 20% of the samples (Table 4). Of the 102 samples, 43 samples did not show loss for any of the tested markers. If we assume that false positive tests are not possible since all patients had a recurrence, the sensitivity of the 12 markers together is 58%. Sensitivity according to grade of the primary tumor is given in File S1. The specificity of the test is per definition 100% because all urines were associated with a recurrent tumor. If we selected urines from those patients whose primary tumor had LOH for at least 1 marker, sensitivity for detection of the recurrence in urine DNA increased to 63%. When combined with cytology data, this sensitivity increased to 80% (File S2).

**Table 4:** Results of the microsatellite analysis of pre-TUR urine samples.

	Total	Het%	LOH/AI%
D8S1109	102	64	7
D8S1125	102	72	9
D8S1130	102	82	10
D9S252	102	79	23
D9S299	101	73	20
D9S304	102	84	22
D9S752	101	84	27
D9S1118	102	88	19
D11S1981	101	84	9
D11S1999	102	88	10
D17S969	101	71	10
G10693	102	74	19

## Discussion

In this study we describe an approach to select microsatellite markers for determining copy number and tumor-associated LOH that have an excellent technical performance. The approach was previously used by Frigerio et al., who implemented marker-specific thresholds by assessing normal DNA from blood and control tissue [1]. However, our approach differs in that we preselected our markers for constant allele ratios thereby enhancing accuracy and by the fact that we selected tri- and tetranucleotide repeats that have no or few stutter peaks. By upfront assessing the lower and upper cut off values based on an analysis of control DNAs, the need to compare patient samples with corresponding blood is therefore avoided. This selection procedure can be applied to any tumor type. This approach also facilitates retrospective analysis of archival tumor samples for allelic imbalance.

We examined the amount of input DNA and the optimal number of PCR cycles. The amount of input DNA is important because too low concentrations may result in preferential amplification of one of the two alleles leading to false positive LOHs [33]. Microsatellite markers are ideal for determining loss or amplification of genomic regions on FFPE-derived DNA because both alleles of a marker will be similarly affected by the quality of the DNA (length) provided that the difference in length between alleles is not too great. Microsatellite analysis is cheap with costs in the order of about 1 euro per assay. For a panel of 10 markers, costs, including DNA isolation, would amount to less than 15 euros. A disadvantage is that microsatellites are not suitable for multiplexing. In our experience this always leads to inefficient amplification of some of the markers and this results in larger standard deviations in duplicate experiments.

Losses on chromosomes 8, 9, 10, 11 and 17 are frequent in bladder cancer and can be detected by microsatellite analysis. This study determines the potential of bladder cancer detection by microsatellite analysis on a set of urine samples collected before transurethral resection of the accompanying tumor (pre-TUR urine). The establishment of marker-specific threshold values based on measurements of allele ratios in urine samples from 106 healthy individuals has allowed us to define the specificity of the method for the subsequent study. Since the individually determined threshold values guaranteed optimal specificity for each of the markers analyzed, we interpreted an LOH at 1 single locus already as indicative for the presence of tumor cells. Applying this rule, we obtained for pre-TUR urine samples an overall sensitivity of 58% for the detection of recurrent tumors and 63% when patients were stratified for LOH in their primary tumor. This sensitivity is comparable to the sensitivity that we previously found with the first set of microsatellite markers even though the new panel of markers was specifically designed to cover for those genomic regions that display LOH in non-muscle invasive bladder tumors (NMIBC). Diagnostic accuracy for the model with all 12 markers was 73% as determined by AUC (Area under the curve). This accuracy was still 73% when only six markers were tested (D9S252, D9S752, D9S304, D8S1125, D8S1130, G10693). With this selection, we were able to identify 95% (56 of 59) of all samples showing LOH when tested for all 12 markers. The main advantage of using a smaller selection of microsatellite markers is, next to a reduction in costs, the reduction of the amount of input DNA needed, making this assay also accessible for those samples where only a very limited amount of tissue is available.

In other studies we used mutation analysis of the *FGFR3* gene in order to diagnose recurrent tumors [34] [35]. *FGFR3* mutations are found in 60–70% of NMIBC and hence provide an ideal tool for surveillance of patients since the mutation assay is 100% specific. Sensitivity, however, depends on the presence of sufficient tumor cells in urine and this is also a caveat for the microsatellite assay. Sensitivity increases when multiple urine samples are analyzed. With a sensitivity of 50% analyzing 2 samples would increase the sensitivity to 75% etc. A combination of microsatellite analysis with the markers presented here and the *FGFR3* test is the subject of a longitudinal study on 800 urine samples from 147 patients (Zuiverloon et al., in preparation).

## Supporting Information

File S1 LOH in pre-TUR urine according to Grade of the primary tumor.
(DOCX)

File S2 Comparison of LOH and Cytology on pre-TUR urine samples.
(DOCX)

Table S1 Patient data.
(XLSX)

Table S2 Details of markers not included in the study.
(XLSX)
