# Supplementary material for: Selection of Microsatellite Markers for Bladder Cancer Diagnosis without the Need for Corresponding Blood
Source: PLoS One. 2012 Aug 22;7(8):e43345. doi: 10.1371/journal.pone.0043345 (PMC3425555; doi:10.1371/journal.pone.0043345)
Supplement: File S1 — LOH in pre-TUR urine according to Grade of the primary tumor. (DOCX) [file pone.0043345.s001.docx]

Supplementary File S1. LOH in pre-TUR urine according to Grade of the primary tumor.

|  | | Grade primary tumor | | Total |
| --- | --- | --- | --- | --- |
|  |  | 1 | 2 |  |
| Pre-TUR urine | no LOH | 12 | 30 | 42 |
|  | LOH | 11 | 47 | 58 |
| Total | | 23 | 77 | 100 |
| Sensitivity | | 48% | 61% |  |

|  | LOH% | | |
| --- | --- | --- | --- |
| Marker | Grade 1 | Grade 2 |  |
| D8S1109 | 9 | 7 |  |
| D8S1125 | 4 | 10 |  |
| D8S1130 | 13 | 8 |  |
| D9S252 | 13 | 25 |  |
| D9S299 | 13 | 22 |  |
| D9S304 | 22 | 21 |  |
| D9S752 | 27 | 28 |  |
| D9S1118 | 17 | 18 |  |
| D11S1981 | 9 | 9 |  |
| D11S1999 | 17 | 8 |  |
| D17S969 | 13 | 9 |  |
| G10693 | 13 | 20 |  |
