# Supplementary material for: Selection of Microsatellite Markers for Bladder Cancer Diagnosis without the Need for Corresponding Blood
Source: PLoS One. 2012 Aug 22;7(8):e43345. doi: 10.1371/journal.pone.0043345 (PMC3425555; doi:10.1371/journal.pone.0043345)
Supplement: File S2 — Comparison of LOH and Cytology on pre-TUR urine samples. (DOCX) [file pone.0043345.s002.docx]

Supplementary File S2. Comparison of LOH and Cytology on pre-TUR urine samples.

All urine samples

|  | | cytology for urine samples | | Total |
| --- | --- | --- | --- | --- |
|  |  | tumor cells found | no tumor cells found |  |
| Urine shows LOH | no LOH | 13 | 22 | 35 |
|  | LOH | 24 | 22 | 46 |
| Total | | 37 | 44 | 81 |

Stratified for LOH in primary tumor

|  | | cytology for U2 | | Total |
| --- | --- | --- | --- | --- |
|  |  | tumor cells found | no tumor cells found |  |
| Urine shows LOH | no LOH | 7 | 8 | 15 |
|  | LOH | 18 | 8 | 26 |
| Total | | 25 | 16 | 41 |
